# Patient and stone characteristics associated with surgical intervention in pediatrics

**DOI:** 10.1186/s40697-015-0057-6

**Published:** 2015-06-02

**Authors:** Esther Jun, Peter Metcalfe, Piush J. Mandhane, R. Todd Alexander

**Affiliations:** Department of Surgery, The University of Alberta, Edmonton, AB T6G 1C9 Canada; Department of Pediatrics, The University of Alberta, Edmonton, AB T6G 1C9 Canada

**Keywords:** Pediatrics, Urolithiasis, Kidney stone surgery

## Abstract

**Background:**

The incidence of kidney stones in children is increasing. While guidelines exist for acute surgical intervention, there is limited data to inform the decision as to when to intervene non-urgently.

**Objectives:**

To identify patient and stone characteristics predicting stone surgery in children.

**Design:**

Retrospective chart review.

**Setting:**

Stollery Children’s Hospital, Edmonton, Alberta, Canada from 1990 to 2013.

**Patients:**

Sixty-three children aged 0–18 years old who presented with a total of 142 stones.

**Measurements:**

Patient’s surgical history, demographics, metabolic measures, and stone number, type, and location.

**Methods:**

Univariate and multivariate analysis, controlling for presentation number and individual-level variation by repeated measures analysis were conducted to assess for patient and stone characteristics associated with surgical intervention.

**Results:**

Sixty-five percent (41/63) required surgery during a mean follow-up of 19 months. Stone characteristics associated with surgical intervention by multivariate analysis included larger stone size (>6 mm), and stone composition of calcium oxalate.

**Limitations:**

Single center study with a limited sample size and duration of follow up, thereby limiting predictive power. There were some missing data (*i,e.* stone type was not always available). Despite this, stone type remained significant in multivariate modeling.

**Conclusion:**

Stone size > 6mm and composition with calcium oxalate but not patient age or symptoms associated with presentation predicted surgical intervention. These observations can be used to inform decisions as to whether urolithiasis should be surgically managed electively or observed.

**Electronic supplementary material:**

The online version of this article (doi:10.1186/s40697-015-0057-6) contains supplementary material, which is available to authorized users.

## Abrégé

**Contexte:**

La fréquence des calculs rénaux chez les enfants est en hausse. Bien que des lignes directrices encadrent les interventions chirurgicales urgentes, les données qui permettent d’éclairer la décision sur le moment opportun d’une intervention non urgente sont limitées.

**Objectifs:**

Cerner les caractéristiques des patients et des calculs rénaux qui pronostiquent le recours à la chirurgie chez les enfants.

**Type d’étude:**

Examen rétrospectif des dossiers.

**Contexte:**

L’hôpital pour enfants Stollery, à Edmonton, en Alberta, au Canada, de 1990 à 2013.

**Patients:**

Soixante-trois enfants de 0 à 18 ans, qui présentaient un total de 142 calculs rénaux.

**Mesures:**

Les antécédents chirurgicaux des patients, les données démographiques, les mesures métaboliques, de même que le nombre de calculs, leur type et leur emplacement.

**Méthodes:**

Analyses unidimensionnelles et multidimensionnelles, qui permettent de contrôler le nombre d’occurrences et les variations sur le plan individuel, grâce à une analyse par mesures répétées, et qui visent à cerner les caractéristiques des patients et des calculs rénaux associées au recours à la chirurgie.

**Résultats:**

Soixante-cinq pour cent (41/63) des cas ont requis une opération au cours de la période de suivi moyenne de 19 mois. À la lumière de l’analyse multidimensionnelle, les caractéristiques des calculs rénaux qui ont nécessité une opération comprenaient leur taille importante (de plus de 6 mm), de même que leur composition en oxalate calcique.

**Limites de l’étude:**

L’efficacité prédictive est réduite, étant donné que l’étude se penche sur un échantillon restreint, dans un seul centre, et au cours d’une période de suivi limitée. Certaines données étaient manquantes (c.-à-d. : le type de calculs n’était pas toujours connu). Pourtant, le type de calculs occupait une place importante dans le modèle multidimensionnel.

**Conclusion:**

Une taille de plus de 6 mm, de même que la composition en oxalate calcique, et non pas l’âge du patient, ni les symptômes associés à la présentation, sont associées au recours à une opération chirurgicale. Ces observations peuvent aider à prendre la décision d’intervenir de manière chirurgicale dans les cas d’urolithiases qui ne présentent pas de critères urgents d’intervention.’

## What was known before

Current indications for acute surgical intervention in children presenting with nephrolithiasis include urinary obstruction, infection, solitary kidney or prolonged symptomatic stones greater than 5 mm. To the best of our knowledge there are no clear indications for elective surgical intervention for childhood urolithiasis.

## What this adds

Our multivariate analysis found that stone size greater than 6 mm and stones composed of calcium oxalate, but not patient age, predicted surgical intervention. This data can help inform a decision as to when to electively remove a stone.

## Background

A recent assessment of the incidence of kidney stones in children reports 50 cases per 100, 000 children [1]. The majority of these stones are composed of calcium, predominantly calcium oxalate but to a lesser extent calcium phosphate. Much less commonly calculi are composed of urate, cysteine or struvite [2]. Moreover the incidence of childhood urolithiasis is increasing. From 1999 to 2008, the percentage of pediatric emergency department visits for renal stones increased by 86 % [1, 3, 4]. Given this trend, the need to ascertain when surgical intervention for pediatric kidney stones is of significant importance [5].

Surgical interventions for urolithiasis in children include: extracorporeal shock wave lithotripsy (ESWL), ureteroscopy, percutaneous nephrolithotomy, and open pyelolithotomy and simultaneous pyeloplasty [6]. Indications for acute surgical intervention of a child with a kidney stone include: urinary obstruction, infection, solitary kidney or prolonged symptomatic stones greater than 5 mm [7]. However, there is limited evidence to inform when intervention in the absence of the above acute indications is prudent, such as an incidental finding on ultrasound or when investigating a child for painless hematuria [7, 4]. In fact, there is some debate, based on these single center studies, as to whether stones can be observed safely in younger children or if they should be removed surgically upon diagnosis [7, 8].

In pediatrics, stone management should consider the preservation of renal function, minimizing radiation exposure, and consequences of surgical intervention such as ureteral stricture formation [9–11]. Our objective was to ascertain patient and stone characteristics that predict surgical intervention, in order to aid the planning of elective surgery thereby avoiding an acute, often painful admission and surgical intervention.

## Methods

### Study design and population

This study was approved by the University of Alberta Human Research Ethics Board (#Pro00040737). We conducted a retrospective chart review of children, ages 0–18 years, presenting to either Pediatric Nephrology or Urology Clinic at the Stollery Children’s Hospital, Edmonton, Alberta between 1990 and 2013. They were followed from date of diagnosis until either: stone elimination, transfer to adult care, or until their last follow-up visit.

### Variables

Demographic data was collected via chart review and included: age, gender, number of kidneys, date of diagnosis of the patient’s first stone presentation, and metabolic characteristics including: serum creatinine, calcium, calcitriol and urine calcium, citrate, and pH at the patients first stone diagnosis date. Number of stone episodes was recorded. “Number of episodes” was defined as the number of times medical attention was sought for a problem attributed to their stone disease. For each episode for each patient, the laterality and location (parenchyma, calyx, pelvis or ureter) of stone(s), total number of stones, stone size (mm) and type (calcium oxalate, calcium phosphate, uric acid, struvite, cysteine or unknown) was recorded. We documented which type of surgical intervention (ESWL, ureteroscopy, percutaneous nephrolithotomy, pyeloplasty or none) the patient received during each episode and radiographic changes to the stone that occurred with follow up. We recorded the symptoms associated with the presentation for each episode (urinary tract infection, gross or microscopic hematuria, flank pain, or incidental i.e. non-symptomatic). Some episodes had multiple symptoms or presenting complaints.

The primary outcome that we analyzed for was any surgical intervention for a stone (either ESWL, ureteroscopy, percutaneous nephrolithotomy, or open pyelolithotomy and simultaneous pyeloplasty). Serum creatinine was considered elevated if it was 2 standard deviations above the age-adjusted upper-limit of normal for our laboratory (for 0–1 year 44 μmol/L, 2–3 years 53 μmol/L, 4–7 years 60 μmol/L, 8–10 tears 80 μmol/L, 11–12 years 88 μmol/L and 13–18 years 108 μmol/L for males and 98 μmol/L for females). Serum calcium was considered elevated if greater than 2.60 mmol/L and serum calcitriol was considered elevated if >168 pmol/L. Urine calcium was considered elevated if the calcium:creatinine ratio was >0.6 in a patient >1 year old, >1.68 if between 6 and 12 months and >2.24 if < 6 months old. Urine citrate was considered low if it was <1.6 mmol/24h [12].

### Statistical analysis

Demographic characteristics were documented with medians and interquartile ranges or means, as appropriate. We explored variables that have been previously associated with surgical intervention and exposures putatively associated with kidney disease in univariate analysis (t-test or chi-squared). Variables that were significant in univariate analysis (p < 0.05) were considered for inclusion in the multivariate model. Multi-level random-effect logistic regression modeling (xtmelogit; STAT 13.1) was used for all analyses. The primary analysis was based on the individual stone outcome (surgery or not). Clustering of the stone within individual was controlled for by modeling stone as the first level in the analysis and individual as the second level. Clustering of the stone within each episode was controlled for by including episode number as a regressor in all analyses. Statistical significance of 0.05 was used for all tests. Interactions were considered based on their biological plausibility. Interactions were considered important if they were significant (p < 0.05) or if they changed the main effects regression coefficients by 10 % or greater.

## Results

### Patient characteristics

**Fig. 1:**
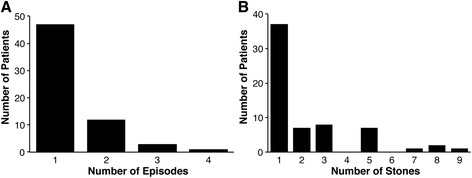
Plot of number of patients by (**a**) number of stones they had or (**b**) number of clinically detectable episodes attributable to their stones

We identified 63 patients between the ages of 0 and 18 years of age who presented to the Stollery Children’s Hospital with urolithiasis. These patients had a total of 142 stones. Patient demographics are detailed in Table 1. Of the 63 patients, 1 patient had a single kidney and always had a surgical intervention for each of their 5 stone episodes (9 stones in total). This individual was excluded from all subsequent analysis. Of the 62 patients with 2 kidneys, 40 patients had at least 1 stone surgery by one of 3 different pediatric urologists. The duration of follow up was 1–96 months. There was no significant difference in mean follow up duration between children who underwent surgery or did not. There was no significant difference in average age for renal stone diagnosis between groups. The average number of stone presentations was 1 in both groups. A plot of the number of stones and stone episodes per patient is presented in Fig. 1.

**Table 1:** Patient Characteristics excluding the 1 patient with a single kidney

	No surgery	Surgery	*P*-value
N patients (%)	22 (35)	40 (65)	
Age at diagnosis, y (SD)	9.6 (5.7)	9.7 (5.3)	0.97
Male	14 (63)	18 (53)	0.72
Duration of follow up, months (SD)	16 (22)	18.6 (25)	0.15
Number of episodes (SD)	1.3 (0.5)	1.4 (0.7)	0.58

### Stone episode characteristics

Of the 133 discrete stones, 76 (57 %) had a surgical intervention. The metabolic and clinical characteristics of the stone episodes are listed in Table 2. None of the metabolic characteristics examined were more prevalent in stone episodes that had surgical intervention. We identified 4 types of presentation at time of stone diagnosis: hematuria, flank pain, urinary tract infection, and incidental stone diagnosis. Flank pain was the most prevalent, which occurred with 86 stones. The next most common presentation was hematuria, presenting with 44 stones. Presentation with a UTI occurred with 27 stones, and there were 18 stones diagnosed incidentally with no other complaints. None of the types of presentation were more common in patients who underwent surgical intervention.

**Table 2:** Characteristics of each stone episode

	No surgery	Surgery	*P*-value
N (%)	57 (40)	76 (57)	
Urine pH (avg)	7.0 (6.7, 7.3)	6.6 (6.5, 6.9)	0.99
High creatinine	4 (3)	6 (5)	0.97
Hypercalciuria	29 (20)	32 (24)	0.46
Hypocitraturia	14 (11)	21 (16)	0.84
Hypercalcemia	8 (6)	3 (2)	0.26
History of UTI’s	48 (34)	61(46)	0.51
Hematuria	11 (8)	33 (25)	0.06
Flank pain	37 (26)	49 (37)	0.62
UTI	14 (10)	13 (10)	0.91
Incidental	7 (5)	11(8)	0.75
Bilateral Stones	5 (14 %)	6 (13 %)	0.85
# of stones (SD)	1.6 (1.0)	1.8 (1.1)	0.43
Stone size at dx (mm) (SD)	4.8 (2.6)	6.7 (4.3)	0.008*

*represents a p value < 0.05

### Influence of stone location and size on surgical intervention

The effect of stone location and size on surgical intervention was next examined (Additional file 1: Table S1). There were 69 stones (52 %) found in the right kidney. The majority of stones were in the calyx (74, 55 %). Thirty-two stones were observed in the ureter, 24 stones were in the pelvis, and 3 stones were in the parenchyma. No specific stone location was associated with an increased rate of surgical intervention, nor did location within the calyx (upper vs lower) predict success of surgical removal. The average size of stone at diagnosis was 4.8 mm in the group that did not have surgery and 6.7 mm in the group that underwent surgery. This difference was statistically significant, p = 0.008. This difference in stone size at last follow-up before an intervention was also significantly different, 4.8 *vs* 7.4 mm, p = 0.005. Consequently stone size, *i.e.* larger than smaller predicted surgical intervention.

### Influence of stone characteristic on surgical intervention

We had stone composition data from 62/133 individual stones (Additional file 1: Table S2). Fifty of these stones were removed surgically while 12 passed spontaneously. Of the total 76 stones surgically removed, 41 were composed of calcium oxalate, 2 were calcium phosphate, 7 were either uric acid, struvite, or cysteine and 26 stones were of unknown composition. Of the 65 stones that did not require surgery, 4 stones were calcium oxalate, 7 stones were calcium phosphate, 53 were of unknown composition, and 1 stone was either uric acid, struvite, or cysteine. We found a statistically significant difference (p < 0.05) in the percentage of stones that did not require surgery for all stone types, except the composite group of uric acid, struvite or cysteine. Calcium oxalate stones were more likely to undergo surgical intervention, while calcium phosphate and unknown stones were less likely to be operated on.

### Multi-variable analysis

Using multi-variate analysis (final complete model presented in supplemental materials, AIC for model = 129), we determined the likelihood of a given variable predicting surgical intervention. Having had a previous stone episode did not predict surgical intervention. When we considered stones of 6 mm and greater vs. smaller stones, there was a 17.1 times greater chance that these stones would undergo surgical intervention. The only other independent variable to predict surgical intervention was stone type. Having a calcium oxalate stone increased the chances of an operative outcome compared to having a calcium phosphate stone (Table 3).

**Table 3:** Multivariate analysis

	Odds ratio of Sx	*P*-value	Confidence interval
Stone size at diagnosis < 6 mm	Reference		
Stone size at diagnosis > 6mm	17.1	0.01	2.3, 128
Calcium phosphate	Reference		
Uric, struvite, cysteine	125	0.05	1.12, 13915
Calcium oxalate	803	0.01	8, 84800
Unknown Stone type	5.6	0.33	0.18, 170

## Discussion

We examined 63 pediatric kidney stone patients presenting with a total of 142 stones to the Stollery Children’s Hospital from 1990 to 2013. Using univariate and multivariate analysis we assessed for either patient or stone characteristics that predicted surgical intervention for the calculi. We found two independent variables predictive of surgical intervention; stone size larger than 6 mm and stone type. Patient age and stone location did not predict surgical intervention. We suggest therefore that stone size and composition be considered in patients presenting with urolithiasis when deciding whether to remove the calculi electively in pediatric patients.

Thus far, factors that increase incidence or recurrence of pediatric urolithiasis have been examined. However, whether these factors are predictive of surgery has not been assessed. Similar analysis to ours in adult populations found that stone size is the most consistent predictor of the need for surgical intervention [13, 14]. To the best of our knowledge, stone type has not been examined as a predictor of surgery. In the absence of pediatric-specific literature, adult guidelines have been extrapolated to pediatric patients such that stone size less than 10 mm can be observed and medically managed as symptoms are tolerated [15]. Given the lack of pediatric literature on predictive factors for surgical intervention, we believe that our findings provide novel insight.

Several recent studies, including a systematic review by Tasian et al., found that the most common metabolic abnormalities associated with repeat renal stone formers are hypercalciuria and hypocitraturia [16, 17, 2]. Pietrow et al. found that children with these metabolic abnormalities were 5-fold more likely to form recurrent or multiple stones [7]. Kovacevic et al. studied specific metabolic risk factors for developing urolithiasis and found hypocitraturia to be the most significant, observing it in approximately 60 % of children with stones [18]. Although we found that metabolic abnormalities, likely leading to stone formation, were common in our cohort, they did not predict surgical intervention for a stone.

We identified four different stone presentations at diagnosis: hematuria, flank pain, urinary tract infection, and incidental. Of these, although not statistically significant (p = 0.06), hematuria was the most indicative of surgical intervention. However, 85–90 % of children with urolithiasis present with hematuria [19, 20]. Given the high prevalence of hematuria in pediatric nephrolithiasis patients, we do not feel that hematuria alone is specific enough of an indicator to warrant surgical intervention.

Van Savage et al. proposed that non-obstructive stones less than 4 mm in diameter should be observed and medically managed [21]. Pietrow et al. had similar findings with only 1 stone greater than 5 mm passing spontaneously [7]. Indeed, we found that the average stone size in the no-surgery group was 4.8 mm while those in the surgery group was significantly larger at 6.7 mm (p = 0008). This result persisted in multivariate analysis. Furthermore, we found that larger stone size at last follow up was also strongly associated with surgical intervention. Our results therefore strongly infer that stones less than 6 mm can be safely observed.

We found that stone composition associates with surgical intervention. The majority of calcium oxalate stones (91 %; p = 0.035) were surgically removed, while 78 % of the calcium phosphate stones passed spontaneously (p = 0.014). Kirejczyk et al. found that calcium oxalate stones showed a strong association with the metabolic risk factors hypercalciuria, oxaluria, magnesuria, and acidic urine - factors predisposing a patient to recurrent stone formation. In contrast, they found that calcium phosphate stones had a lower association with other risk factors including infection and impaction [22]. This suggests that calcium oxalate stones more often warrant surgical intervention due to their risk for causing complications and recurrent formation. Conversely, knowing that an individual is forming calcium phosphate stones supports more conservative management because they were more likely to pass without complications. Although of borderline significance, it was also noted that 7 of the 8 stones composed of uric acid, cysteine, or struvite were surgically removed. This finding is consistent with previous work that found struvite, cysteine and uric acid stones have unique durability and remarkable sizes that make passing the stone spontaneously unlikely [17].

Although we analyzed a significant number of stones in our cohort, this study has several limitations. The single center nature of the study would limit the total number of patients and reduce statistical power in the analysis. We performed much of the analysis at the level of the stone to increase power, however, there was clustering of stone factors within a given patient. We only had one child with a single kidney, which always had surgery, making conclusions on this population difficult. The duration of follow up was on average slightly less than two years, which limits the predictive power of this work. As this was a retrospective chart review we were limited by some missing data. Moreover, some stones that passed spontaneously were not collected, further reducing the number of stones we knew the composition of. Regardless, we have been able to identify factors predicting surgical intervention in children with urolithiasis.

## Conclusions

In summary we examined 133 stones in 62 children with urolithiasis for factors predicting surgical intervention. We found that calcium oxalate stones and stones greater than 6 mm were more likely to require surgical intervention. These findings will help inform decisions as to whether a child should undergo elective stone surgery or conservative observational management.

## Additional file

Additional file 1: Table S1. Stone Location and Size. **Table S2.** Stone Composition. **Table S3.** Complete multi-level random-effect logistic regression model.
