# Supplementary material for: Patient and stone characteristics associated with surgical intervention in pediatrics
Source: Can J Kidney Health Dis. 2015 Jun 2;2:22. doi: 10.1186/s40697-015-0057-6 (PMC4451733; doi:10.1186/s40697-015-0057-6)
Supplement: Additional file 1: Table S1. — Stone Location and Size. Table S2. Stone Composition. Table S3. Complete multi-level random-effect logistic regression model. [file 40697_2015_57_MOESM1_ESM.docx]

**Supplemental Material**

**Supplemental Table 1. Stone Location and Size**

|  | **No Surgery** | **Surgery** | **P-value** |
| --- | --- | --- | --- |
| **N, (%)** | 57 (43) | 76 (57) |  |
| **Right kidney presentation** | 27 (40) | 42 (32) | 0.90 |
| **Parenchyma #** | 0 | 3 (2) | 0.22 |
| **Calyx #** | 39 (27) | 35 (26) | 0.28 |
| **Pelvis #** | 8 (6) | 16 (12) | 0.72 |
| **Ureter #** | 10 (7) | 22 (17) | 0.16 |
| **Size of stone at last F/U (mm)** | 4.8 (4.2, 5.6) | 7.4 (6.9,9.1) | 0.005* |

# represents stone location at initial presentation

**Supplemental Table 2. Stone Composition**

|  | **Passed** | **Surgical removal** | **P-values** |
| --- | --- | --- | --- |
| **N, (%)** | 65 (46) | 76 (54) |  |
| **Calcium oxalate** | 4 (3) | 41 (29) | 0.035* |
| **Calcium phosphate** | 7 (5) | 2 (1) | 0.014* |
| **Uric acid, struvite, cysteine** | 1 (1) | 7 (5) | 0.36 |
| **Unknown** | 53 (37) | 26 (18) | 0.002* |

**Supplemental Table 3.** Complete multi-level random-effect logistic regression model

| **Surgical intervention** | **Odds Ratio** | **[95% Conf. Interval]** | | **P>z** |
| --- | --- | --- | --- | --- |
| Stone diameter < 6 mm | Reference |  |  |  |
| Stone diameter >= 6 mm | 17.10 | 2.28 | 128.38 | 0.01 |
|  |  |  |  |  |
| **Stone type** |  |  |  |  |
| Calcium phosphate | Reference |  |  |  |
| Calcium oxalate | 802.63 | 7.60 | 84810.58 | 0.01 |
| Uric acid, struvite, cysteine | 124.85 | 1.12 | 13915.07 | 0.05 |
| Unknown | 5.59 | 0.18 | 171.60 | 0.33 |
|  |  |  |  |  |
| **Presentation No.** |  |  |  |  |
| 1^st^ presentation | Reference |  |  |  |
| 2^nd^ presentation | 1.39 | 0.29 | 6.64 | 0.68 |
| 3^rd^ presentation | 0.12 | 0.00 | 4.66 | 0.26 |
| Constant | 0.02 | 0.00 | 0.85 | 0.04 |

| Random-effects Parameters | Estimate | [95% Conf. Interval] | |
| --- | --- | --- | --- |
| Cluster (individual patient) |  |  |  |
| sd(_cons) | 2.19 | 1.05 | 4.56 |

LR test vs. logistic regression (chibar^2^): p-value =0.0003
